# Determinants of Use of the Care Information Exchange Portal: Cross-sectional Study

**DOI:** 10.2196/23481

**Published:** 2021-11-11

**Authors:** Ana Luisa Neves, Katelyn R Smalley, Lisa Freise, Paul Harrison, Ara Darzi, Erik K Mayer

**Affiliations:** 1 Patient Safety Translational Research Centre Institute of Global Health Innovation Imperial College London United Kingdom; 2 Center for Health Technology and Services Research Faculty of Medicine University of Porto Porto Portugal; 3 Department of Community Medicine Health Information and Decision University of Porto Porto Portugal; 4 Imperial National Health Service Healthcare Trust London United Kingdom

**Keywords:** patient portals, electronic health records, patient participation

## Abstract

**Background:**

Sharing electronic health records with patients has been shown to improve patient safety and quality of care. Patient portals represent a convenient tool to enhance patient access to their own health care data. However, the success of portals will only be possible through sustained adoption by its end users: the patients. A better understanding of the characteristics of users and nonusers is critical for understanding which groups remain excluded from using such tools.

**Objective:**

This study aims to identify the determinants of the use of the Care Information Exchange, a shared patient portal program in the United Kingdom.

**Methods:**

A cross-sectional study was conducted using a web-based questionnaire. Information collected included age, gender, ethnicity, educational level, health status, postcode, and digital literacy. Registered individuals were defined as having had an account created in the portal, independent of their actual use of the platform; users were defined as having ever used the portal. Multivariate logistic regression was used to model the probability of being a user. Statistical analysis was performed in R and Tableau was used to create maps of the proportion of Care Information Exchange users by postcode area.

**Results:**

A total of 1083 participants replied to the survey (186% of the estimated minimum target sample). The proportion of users was 61.58% (667/1083). Among these, most (385/667, 57.7%) used the portal at least once a month. To characterize the system’s users and nonusers, we performed a subanalysis of the sample, including only participants who had provided at least information regarding gender and age. The subanalysis included 650 individuals (389/650, 59.8% women; 551/650, 84.8% >40 years). Most participants were White (498/650, 76.6%) and resided in London (420/650, 64.6%). Individuals with a higher educational degree (undergraduate and professional, or postgraduate and higher) had higher odds of being a portal user (adjusted odds ratio [OR] 1.58, 95% CI 1.04-2.39 and OR 2.38, 95% CI 1.42-4.02, respectively) compared with those with a secondary degree or below. Higher digital literacy scores (≥30) were associated with higher odds of being a user (adjusted OR 2.96, 95% CI 2.02-4.35). Those with a good overall health status had lower odds of being a user (adjusted OR 0.58, 95% CI 0.37-0.91).

**Conclusions:**

This work adds to the growing body of evidence highlighting the importance of educational aspects (educational level and digital literacy) in the adoption of patient portals. Further research should not only describe but also systematically address these inequalities through patient-centered interventions aimed at reducing the digital divide. Health care providers and policy makers must partner in investing and delivering strategic programs that improve access to technology and digital literacy in an effort to improve digital inclusion and reduce inequities in the delivery of care.

## Introduction

### Background

A growing body of evidence supports providing patients access to their electronic health records to improve several aspects of quality of care, including patient safety [1-3], patient-centeredness [4,5], and effectiveness [3]. Patient portals are currently recognized as a promising mechanism for improving health care data sharing with patients. Patients may use portals for a range of purposes, including entering, retrieving, or sharing their health care information, communicating with health care providers, and self-managing their health [6]. The use of patient portals can improve health outcomes (eg, in the case of type 2 diabetes) [7,8], increase patient satisfaction [9], improve medication safety and adherence [10-12], and improve communication between the patient and the health care provider [9-13].

The success of patient portals and the subsequent achievement of their proposed benefits will only be possible through sustained adoption by its end users: the patients. However, questions remain about how health care providers and policy makers can encourage sustainable adoption by patients without exacerbating the pre-existing digital divide or widening discrepancies in the delivery of care [14]. In fact, despite the increasing implementation of patient portals by health care institutions and governments worldwide, adoption by patients has remained slower than expected [15,16]. A meta-analysis published by Fraccaro et al [17], including 40 studies, showed an overall mean adoption rate of 52% (95% CI 42%-62%). However, the authors emphasize that the evaluation of adoption in clinical practice may have different results from those obtained in randomized controlled trials [17].

Several individual and sociotechnical factors have been suggested to affect portal adoption, such as age, health status, educational level, and patient activation (ie, the knowledge, skills, and confidence a person has in managing their own health and care) [18-20]. Although there is some evidence of higher adoption by those with poorer health status [21,22] and higher educational level [20], there is mixed evidence about the impact of age [21,23] and patient activation [24,25]. Technology-related factors may also play an important role, with higher digital health literacy, better portal design, and higher perceived usefulness and ease of use being potentially associated with a positive impact [26].

In 2015, Patients Know Best teamed up with Imperial College National Health Service Healthcare Trust to roll out the *Care Information Exchange* (CIE) across North West London the largest shared patient portal program in the United Kingdom, hosting records of more than 2.3 million people living in North West London [27]. The CIE collects data from hospitals and general practitioners’ practices in North West London and 15 other hospitals outside of North West London, including Birmingham, Bristol, Liverpool, Manchester, Scotland, and Wales [27]. The CIE was the first to introduce *mass registration*, enabling people to sign up and access their health record at scale and with speed in a number of ways: either by speaking to a member of staff, using the kiosk check-in screen commonly found in hospital outpatient waiting rooms, or by letter of invitation to their home. The CIE contains patient information, including appointment details, test results, care plans, discharge summaries, clinical letters, and information on medications. If a patient’s primary care practice has signed up, data such as allergies, medications, and diagnoses will also be visible to them. Patients may access their records whenever they wish to review information or when notified about new information, such as available test results [3].

### Objectives

This study aims to characterize individuals registered with the CIE and explore the differences between users and nonusers in terms of their demographic, geographic, health status, and educational characteristics (ie, educational level and digital health literacy) and motivation to be involved in their own health care (as a proxy measure for patient activation), thus identifying the main determinants of use of the portal. Our hypothesis is that the users’ characteristics described above can affect the adoption of CIE. This is key to understanding barriers to adoption as well as understanding which groups remain underserved or excluded from using patient portals, which is critical for future patient-centered digital health care delivery.

## Methods

### Study Design, Participants, and Data Collection

We conducted a cross-sectional study using an anonymous web-based questionnaire presented by Qualtrics.

Patients registered with the CIE portal and who had logged in at least once during the study period (n=27,411) were invited to follow the link to complete the survey. There were no specific exclusion criteria; however, patients needed to be ≥18 years to register to use the portal. This link contained general information about the purpose of the survey, and informed consent was obtained at the beginning of the survey. Considering this population, a confidence level of 95% and a margin of error of 5%, the minimum sample size to ensure representativeness was calculated as n=379 respondents. The survey was open for completion between July 1, 2018, and July 1, 2019. No patient identifiers were collected. Information collected included age, gender, ethnicity, educational level, postcode (first part), digital literacy, health status, and motivation to be involved in their own health care (as a proxy for patient activation).

### Measurements

Age was categorized into age bands (<30, 31-40, 41-50, 51-65, ≥65), and ethnicity was categorized as White or Black, Asian, and minority. The first part of the postcode was categorized as London’s official postal district for descriptive purposes [28]. For the univariate and multivariate analyses, owing to the highly skewed distribution toward West and North West London, postcode areas were categorized as West London, North West London, other London, or other.

Digital literacy was assessed using the eHealth Literacy Scale (eHEALS), developed by Norman and Skinner [29]. This tool identifies six core skills or literacies: (1) traditional literacy, (2) health literacy, (3) information literacy, (4) scientific literacy, (5) media literacy, and (6) computer literacy. On the basis of these core literacies, the eHEALS tool assesses consumers’ knowledge, comfort, and perceived skills at finding, evaluating, and applying eHealth information to health problems. The eHEALS tool uses a 5-point Likert scale (1-strongly disagree and 5-strongly agree), with a score ranging from 8 to 40, with a higher score indicating higher literacy.

Overall health status was assessed via a multiple-choice question (“How good do you think your health is?” with possible responses: “Very good,” “Somewhat good,” “Neither good nor poor,” “Somewhat poor,” and “Very poor”). Motivation to be involved in their own care was similarly assessed via multiple-choice questions (“In general, how motivated to be involved in your health care are you?” with possible responses: “A little,” “A moderate amount,” “A lot,” and “Very much”).

Registered individuals were defined as having had an account created in the CIE portal, independent of their actual use of the platform. *Users* and *nonusers* were defined as individuals having answered “Yes” or “No,” respectively, to the question “Have you ever used CIE?” Those who answered “Yes” (ie, users) were also asked about their frequency of use (“How often do you use CIE?” with response options as follows: “Less than once a month,” “Once a month,” “Once a week,” and “Twice a week or more”).

### Data Analysis

Mean and SD were calculated for continuous variables, and proportions and counts were calculated for categorical variables. Univariate logistic regression modeled the odds of being a user as a function of each individual predictor. The resulting coefficients, expressed as log (odds) ratios, were transformed into crude odds ratios (ORs) with a 95% CI.

Multivariate logistic regression was used to model the probability of being a user as a function of age, gender, educational level, digital literacy (categorical variables), and overall health status. The variables were chosen for multivariate analysis through automated, backward stepwise elimination. With this procedure, all variables of interest are included in the first iteration of the model and removed one by one, starting with the ones for which elimination would improve the model fit most and ending the process when removing an additional variable worsens the model fit. Model quality comparisons were conducted using the Akaike information criterion [30]. Basic demographic variables (age and gender) were inputted as forced-in covariates in the multivariate analysis. Adjusted ORs with 95% CI were calculated.

Statistical analyses were conducted in RStudio, using the *plyr*, *dplyr*, *ggplot2*, and *car* packages. Tableau software was used to create maps of the total number of participants and the proportion of CIE users by postcode area.

### Ethics

The study was approved as a Service Evaluation at Imperial College Healthcare National Health Service Trust (Registration Number: 296/2018).

## Results

### Participants’ Characteristics

The survey link was shared with a total of 27,411 patients that logged at least once (ie, were accredited to use the system) between July 1, 2018, and July 1, 2019. A total of 1083 subjects replied to the survey (186% of the estimated target sample). The proportion of users was 61.58% (667/1083), and among these, more than half (385/667, 57.7%) used the portal at least once a month. Self-identified users and nonusers of CIE were more likely to provide demographic information (for age: 251/667, 37.6% and 152/416, 36.5%, respectively; for gender: 246/667, 36.9%) and 36.6% (152/416). Of them, 650 participants provided information regarding their gender and age, and we limited the analysis to these individuals (+71.5% of the estimated target sample).

**Figure 1:**
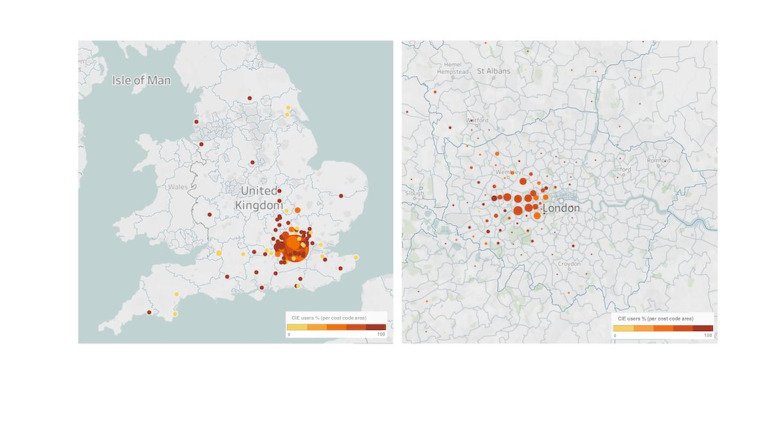
Geographic location overview. General overview of England (left) and Central London (right, representing 64.7% of the subjects). Circle size represents the total number of respondents per postcode area, and color code represents the percentage of Care Information Exchange users per postcode area. The right-side image shows the stronger representation of North West London in the sample. CIE: Care Information Exchange.

In the subanalysis of patients who provided basic characteristics regarding gender and age category, 59.8% (389/650) were women, and 84.8% (551/650) were ≥40 years. Most participants were White (498/650, 76.6%) and resided in London (651/1006, 64.7%). Among them, 55.9% (363/651) were from North West London. A more detailed overview of the distribution of participants by postcode area is provided in Figure 1. The mean literacy score assessed by the eHEALS tool was 31.5 (SD 7.9), and 22.3% (145/651) had a postgraduate degree or higher. Most participants considered themselves very motivated to be involved in their own care (374/651, 57.5%), and 41.8% (272/651) considered themselves to have a good or very good health status. A full description of the analyzed sample and the characteristics of the nonuser and user groups is provided in Table 1.

**Table 1:** Characteristics of the participants according to their use of the system (N=650).

Characteristics				Nonusers (n=205)		Users (n=447)		Total (N=650)
**Gender, n (%)**								
	Female		113 (55.1)		276 (61.7)		389 (59.8)	
	Male		91 (44.4)		167 (37.4)		258 (39.7)	
	Other		1 (0.5)		2 (0.4)		3 (0.5)	
	No response		0 (0)		0 (0)		0 (0)	
**Age band (years), n (%)**								
	<30		9 (4.4)		22 (4.9)		31 (4.8)	
	31-40		20 (9.8)		48 (10.7)		68 (10.5)	
	41-50		23 (11.2)		62 (13.9)		85 (13.1)	
	51-65		72 (35.1)		166 (37.1)		238 (36.6)	
	≥65		81 (39.5)		147 (32.9)		228 (35.1)	
	No response		0 (0)		0 (0)		0 (0)	
**Ethnicity, n (%)**								
	BAME^a^		34 (16.6)		75 (16.8)		109 (16.8)	
	White		155 (75.6)		343 (76.7)		498 (76.6)	
	Other		16 (7.8)		22 (4.9)		38 (5.8)	
	No response		0 (0)		5 (1.1)		5 (0.8)	
**Geographic location, n (%)**								
	**London**							
		East	2 (0.9)		2 (0.5)		4 (0.6)	
		East Central	0 (0)		0 (0)		0 (0)	
		North	0 (0)		7 (1.6)		7 (1.1)	
		North West	15 (7.3)		40 (8.9)		55 (8.5)	
		South East	0 (0)		5 (1.1)		5 (0.7)	
		South West	20 (9.8)		28 (6.3)		48 (7.4)	
		West	96 (46.8)		203 (45.4)		299 (46)	
		West Central	1 (0.5)		1 (0.2)		2 (0.3)	
	Other		62 (30.2)		146 (32.7)		208 (31.8)	
	No response		9 (4.4)		13 (2.9)		22 (3.5)	
**Educational degree, n (%)**								
	Secondary school or below		75 (36.6)		118 (61.1)		193 (29.7)	
	Undergraduate or professional degree		77 (37.6)		180 (40.3)		257 (39.5)	
	Postgraduate or higher		33 (16.1)		112 (25.1)		145 (22.3)	
	No response		20 (9.8)		35 (7.8)		55 (8.5)	
Digital literacy (eHEALS^b^ score), mean (SD)				28.4 (8.1)		32.9 (7.4)		31.5 (7.9)
**Overall health status, n (%)**								
	Good or very good		95 (46.3)		177 (39.6)		272 (41.8)	
	Neither good nor poor		55 (26.8)		106 (23.7)		161 (24.8)	
	Poor or very poor		55 (26.8)		162 (36.2)		217 (33.3)	
	No response		0 (0)		0 (0)		0 (0)	
**Motivation to be involved in own care, n (%)**								
	Not very much		7 (3.4)		6 (1.34)		13 (2)	
	A moderate amount		40 (19.5)		43 (9.6)		83 (12.7)	
	A lot		61 (29.8)		116 (25.9)		177 (27.2)	
	Very much		96 (46.8)		278 (62.2)		374 (57.5)	
	No response		1 (0.5)		2 (0.5)		3 (0.5)	

^a^BAME: Black, Asian, and minority ethnic.

^b^eHEALS: eHealth Literacy Scale.

### Differences Between Users and Nonusers

The characteristics of both users and nonusers were explored using univariate logistic regression (crude ORs) and a logistic regression model with predictors (adjusted ORs). The differences between groups are shown in Table 2.

Crude ORs showed that individuals with a higher educational degree (undergraduate or professional or postgraduate or higher) had higher odds of being a portal user (crude OR 1.48, 95% CI 1.00-2.20 and crude OR 2.15, 95% CI 1.33-3.05, respectively) than those with a secondary degree or below. Higher digital literacy scores (>30) were also associated with higher odds of being a user (crude OR 2.90, 95% CI 2.06-4.11) and those that reported being “very much motivated to be involved in their own care” (crude OR 3.38, 95% CI 1.10-10.3). Participants with a good overall health status had lower odds of being users (crude OR 0.63, 95% CI 0.43-0.94).

Adjusted ORs represent the multivariate analysis of the predictors of CIE use. Initially, all variables were included in the multivariate model, and backward stepwise elimination was used to select the best-fit model. Digital literacy, education, and health status remained in the naive best-fit regression, and gender and age were reinputted as forced-in covariates, as previously described in the *Methods* section. Sensitivity analyses showed minimal differences as a result of their inclusion or exclusion. All covariates that were statistically significant in the naive model remained, and no additional variables gained significance.

Adjusted ORs showed that individuals with a higher educational degree (undergraduate or professional or postgraduate or higher) had higher odds of being a portal user (adjusted OR 1.58, 95% CI 1.04-2.39 and adjusted OR 2.38, 95% CI 1.42-4.02, respectively) than those with a secondary degree or below. Higher digital literacy scores (≥30) were also associated with higher odds of being a user (adjusted OR 2.96, 95% CI 2.02-4.35). Those with a good overall health status had lower odds of being a user (adjusted OR 0.58, 95% CI 0.37-0.91).

A significant association was found with *increased motivation to be involved in own care* (crude OR) for those very motivated to be involved in their own care is 3.38 (95% CI 1.10-10.3). However, it was not possible to explore the effect in multivariate analysis as the variable was removed from the best-fit model as part of the stepwise backward elimination procedure. No significant associations were found with age, gender, ethnicity, or geographic location.

**Table 2:** Characteristics of users according to their input with crude and adjusted odds ratios (ORs; N=650).

Characteristics		Nonadjusted model^a^			Adjusted model^b^	
		Crude OR (95% CI)	*P* value	Adjusted OR (95% CI)		*P* value
**Gender**						
	Female	Reference	N/A^c^	Reference		N/A
	Male	0.75 (0.54-1.05)	.01	0.92 (0.624-1.35)		.67
	Other	0.81 (0.07-9.12)	.87	0 (0-infinity)		.98
**Age band**						
	<30	Reference	N/A	Reference		N/A
	31-40	0.98 (0.39-2.50)	.97	0.63 (0.22-1.76)		.37
	41-50	1.10 (0.44-2.74)	.83	0.88 (0.32-2.40)		.80
	51-65	0.94 (0.41-2.15)	.89	0.85 (0.34-2.12)		.73
	≥65	0.74 (0.33-1.69)	.47	0.65 (0.26-1.65)		.37
**Ethnicity^d^**						
	White	Reference	N/A	—^e^		—
	BAME^f^ or other	0.88 (0.59-1.33)	.55	—		—
**Geographic location^d^**						
	West London	0.94 (0.65-1.36)	.74	—		—
	North West London	1.19 (0.62-2.29)	.41	—		—
	Other London	0.85 (0.47-1.53)	.59	—		—
	Other	Reference	N/A	—		—
**Educational degree**						
	Secondary or below	Reference	N/A	Reference		N/A
	Undergraduate or professional	1.48 (1.00-2.20)	.049	1.58 (1.04-2.39)		.001
	Postgraduate or higher	2.15 (1.33-3.50)	.002	2.38 (1.42-4.02)		.03
**Digital literacy**						
	Literacy score <30	Reference	N/A	Reference		N/A
	Literacy score ≥30	2.90 (2.06-4.11)	<.001	2.96 (2.02-4.35)		<.001
**Overall health status**						
	Poor	Reference	N/A	Reference		N/A
	Neutral	0.65 (0.42-1.02)	.06	0.73 (0.45-1.20)		.21
	Good	0.63 (0.43-0.94)	.02	0.58 (0.37-0.91)		.02
**Motivation to be involved in own care^d^**						
	Not very much	Reference	N/A	—		—
	A moderate amount	1.25 (0.39-4.05)	.17	—		—
	A lot	2.22 (0.71-6.89)	.71	—		—
	Very much	3.38 (1.10-10.3)	.03	—		—

^a^Crude odds ratios calculated from univariate logistic regression, where the probability of being a user was modeled.

^b^Logistic regression model with predictors: age, gender, education level, digital literacy, and health status.

^c^N/A: not applicable.

^d^These variables were removed from multivariate analysis using a stepwise backward elimination procedure.

^e^Not available.

^f^BAME: Black, Asian, and minority ethnic.

## Discussion

### Principal Findings

Participants with an undergraduate or professional degree were 58% more likely to use the portal than those with secondary education or below (adjusted OR 1.58, 95% CI 1.04-2.39), and those with a postgraduate degree were more than 2 times as likely to use the portal (adjusted OR 2.38, 95% CI 1.42-4.02). People with an eHEALS digital literacy score greater than 30 were nearly three times more likely to be portal users than those with eHEALS scores below 30 (adjusted OR 2.96, 95% CI 2.02-4.35). These results reveal the impact of education and literacy on the adoption of digital technologies and reinforce their role as drivers of patient exclusion.

Participants with good overall health status were about half as likely to have reported using the portal compared with those reporting poor health status (adjusted OR 0.58, 95% CI 0.37-0.91). This reinforces that although the ability to use digital technologies is an important contributor to their adoption, the perceived need for service is equally important. People with poor health will have more incentive to engage with technologies that facilitate their health care than those without health challenges.

No significant associations were found with age, gender, ethnicity, geographic location, or motivation to be involved in their own care. The fact that demographic factors such as these were not strongly associated with use further indicates that ability and need to use digital health management tools are potentially the key drivers of their uptake.

### Comparison With Previous Literature

Our findings are consistent with previous evidence suggesting that portal users, compared with nonusers, are more often highly educated and have higher eHealth literacy levels [31].

The spread and scale of digitally enabled care are happening fast—in fact, faster than our ability to ensure that all patient groups have the basic digital literacy tools to fully exploit its potential. The educational level seems to be an independent predictor of portal use; in an inpatient study, after adjusting for age, gender, race and ethnicity, immigration status, educational attainment, and employment status, those without an education degree had higher odds of never logging on to the portal [20].

Previous studies have also found that patients with higher eHealth literacy levels are more likely to be portal users [31,32]. Importantly, people’s self-perceived skills to use web-based information actually have an impact on their health and the quality of care received, and a lack of such skills may result in adverse health outcomes [33,34]. According to Holt et al [35], information about patients’ health literacy may provide a better understanding of patients’ reasons for not using digital health services rather than sociodemographic data.

The educational level seems to be an independent predictor of portal use; in an inpatient study, after adjustment for age, gender, race and ethnicity, immigration status, educational attainment, and employment status, those without an education degree had higher odds of never logging on to the portal [20]. Previous studies also found that patients with higher eHealth literacy have a higher likelihood of being portal users [31,32]. Similar findings were reported by Holt et al [35], suggesting that information about patients’ health literacy may provide a better understanding of patients’ reasons for not using digital health services rather than sociodemographic data.

The association between having a good overall health status and a lower likelihood of being a portal user has also been documented in previous studies. People with disabilities, chronic conditions, and frequent use of health care services (and caregivers of elderly parents or children) tend to be associated with higher patient portal interest and use [19,22]. In this study, we did not find any significant associations with age, gender, ethnicity, or geographic location. The association between age and portal use has been inconsistently reported, and although some studies have suggested that elderly people use portals less often [19,22,23], others did not find a significant effect [36]. Mixed results have also been found regarding gender differences [33,34]. It has been previously suggested that ethnic minorities use patient portals less often [37]. However, a study evaluating disparities in enrollment and use of a patient portal concluded that although minority patients were less likely to register to use a patient portal, there were no racial and ethnic disparities in the use of the patient portal among enrollees, suggesting that the digital divide may be particularly important at enrollment, rather than in continued use (ie, postenrollment) [21]. It is likely that the association between ethnicity and portal use results from a complex relationship modeled by a range of sociodemographic, economic, and educational variables. In this study, ethnicity was not, per se, an independent predictor of portal use.

A significant association was found between portal use and patient activation (expressed as the subjective motivation to be involved in one’s own care), but significance did not remain in multivariate analyses—in fact, this variable was removed from the best-fit model as part of the stepwise backward procedure. A few studies exploring this aspect have found inconsistent results: while one study found slightly higher patient activation measure scores in portal users [24], others found no significant associations between patient activation measure levels and portal log-in [25].

### Strengths and Limitations

This study had several strengths. The sample size was 75% higher than the estimated minimum sample size to ensure representativeness and adequate statistical power. A comprehensive set of characteristics was collected and analyzed at the individual level, allowing us to explore not only the classic demographic factors (age, gender, ethnicity, and educational level) but also important variables such as overall health status, motivation to self-manage, and health literacy (using a validated tool). The high response rate and the overall large sample size contribute to the robustness of these findings.

Some limitations of this study should also be acknowledged. First, it must be noted that, although we achieved the minimum sample size required, the overall response rate was low, which has important considerations for generalizations about which determinants drive portal adoption. Intrinsically to the study design, a range of selection biases cannot be excluded. Although web-based surveys are a well-accepted method for data collection, they induce a selection bias by excluding less tech-savvy individuals, individuals with less digital literacy, with less consistent access to the internet, and therefore those who are less likely to adopt patient portals. Using an exclusively web-based recruitment strategy also introduces an additional selection bias, but unfortunately, we were not able to email participants directly because of information governance limitations. In addition, both users and nonusers were registered at the portal, and therefore our results highlight potential determinants of use among registered users and not general determinants of initial engagement with the portal. This needs to be considered in any attempt to perform external generalizations. Although this study aimed to identify the determinants of use between those that had already registered (not the determinants to engage or register with a portal in the first instance), future research should also address determinants of initial engagement (ie, register with a portal in the first instance).

In this study, patient portal use was patient-reported; therefore, a potential information bias could also be present. As an alternative, patient log-in can be used to measure portal use [34]; however, this approach lacks contextual information.

It is also important to note that participants included in this study were predominantly from a specific geographic location (North West London); therefore, these results need to be carefully interpreted in any attempt to perform external generalizations (ie, to other geographic locations, populations, or health care systems). In future work, it would be important to evaluate not only the geographic location of the users but also whether users live in an urban, rural, or mixed setting, given the variation in accessibility (ie, internet access and connectivity options) among those.

Finally, this study aimed to evaluate the impact of individual factors. However, there are a plethora of sociotechnical factors (including factors such as social determinants, portal design, and communication strategies) that may equally influence adoption rates and the impact that is important for evaluation in future research.

### Conclusions

This work adds to the growing body of evidence highlighting the importance of educational aspects (educational level and digital literacy) for sustainable implementation and use of patient-facing electronic health record portals. To ensure that all patients are able to benefit from patient portals, it is critical that we move from identifying disparities in portal use to systematically addressing them through patient-centered interventions that reduce the digital divide.

Further research evaluating the impact of interventions to improve portal use must therefore explore the effect on potential disparities in use, addressing the impact on patients with a low educational level, poor access to technology, or lack of ability or confidence to use it for health-related purposes.

Equally, portal use can be improved by co-designing portals with patients, incorporating user-centered design techniques, and ensuring that a diverse group of potential users is included in the process. In particular, involving older persons and those with lower general health literacy and digital health literacy in digital development can provide important insights into the barriers experienced by these typically excluded groups and co-design strategies to overcome them [38].

Therefore, it is critical to ensure that health care providers and policy makers align across sectors, investing and delivering strategic programs that improve access to technology and digital literacy, in an effort to improve digital inclusion and reduce inequities in the delivery of care.

## Abbreviations

CIE: Care Information Exchange
eHEALS: eHealth Literacy Scale
NIHR: National Institute for Health Research
OR: odds ratio
